# Acute Effects of a Week of Stochastic‐Random Whole‐Body Vibration on Manual Ability Performance in Healthy Subjects: A Preliminary Pilot Study

**DOI:** 10.1155/bmri/7662370

**Published:** 2025-09-23

**Authors:** Isabel Mª. Alguacil Diego, Rosa Mª. Martínez Piédrola, Ángela Aguilera Rubio, Miguel Gómez Alguacil, Francisco Molina Rueda

**Affiliations:** ^1^ Department of Physical Therapy, Occupational Therapy, Rehabilitation and Physical Medicine, Faculty of Health Sciences, Rey Juan Carlos University, Alcorcón, Madrid, Spain, urjc.es; ^2^ Motion Analysis, Biomechanics, Ergonomics and Motor Control Laboratory (LAMBECOM), Faculty of Health Sciences, Rey Juan Carlos University, Alcorcón, Madrid, Spain, urjc.es; ^3^ Research Group in Evaluation and Assessment of Capacity Functionality and Disability, Universidad Rey Juan Carlos, Alcorcón, Madrid, Spain, urjc.es; ^4^ Armed Forces medical service, Agoncillo military base, Ministry of Defence, La Rioja, Spain

**Keywords:** manual skills, motor dexterity, rehabilitation, vibrating platform, whole-body vibration

## Abstract

**Aim:** The aim of the study is to evaluate whether stochastic whole‐body vibration (WBV) can improve dexterity and manipulative capacity in healthy individuals using a conventional squat position over a short period (1 week).

**Introduction:** Many pathologies specifically affect fine motor dexterity. Numerous therapies are aimed at improving upper extremity functionality. In this context, WBV appears to represent a significant breakthrough in the field of rehabilitation. The mechanical vibration signals activate sensory receptors (muscle spindles), triggering reflex muscle activation like the tonic vibration reflex. The random mechanoreceptor stimulation maintains continuous brain activation, increasing corticospinal excitability.

**Methods:** Thirty‐eight healthy young volunteers were randomized to the WBV group (*N* = 19; 6 men, 13 women) or the control group (*N* = 19; 6 men, 13 women). The subjects in the WBV group performed one series of five consecutive repetitions of 60‐s unsynchronized WBV (Zeptoring, Scisen GmbH, Germany; mechanical vibration of 4 Hz and amplitude of 3 mm) with a 1‐min pause between administrations, three times a week. Baseline and 5 min after the intervention, preferred hand (PH), nonpreferred hand (NPH), both hands (BH), and assembly (A) of the Purdue Pegboard test were performed.

**Results:** Student’s *t*‐test showed a significant advantage in favor of the WBV group for NPH and BH compared to the control group. Among all variables, the NPH showed the most substantial improvement, with an increase of approximately 12% (*p* < 0.02), while BH improved by 8% (*p* < 0.01).

**Conclusions:** Acute low‐frequency WBV in a squat position improves manual dexterity in healthy subjects. Future studies with larger sample sizes and diagnostic methods are needed to support these findings.

**Trial Registration:** ClinicalTrials.gov identifier: NCT03289689

## 1. Introduction

The functionality of the upper extremities (UEs), particularly the hands, is one of the key traits that distinguish humans from other species [1]. Many pathologies specifically affect fine motor dexterity, which is defined as the ability to perform rapid, skillful, controlled movements and manipulate small objects, primarily involving the fingers [2]. Such impairments significantly impact activities of daily living and, consequently, the individual’s quality of life. Occupational performance can be disrupted by a wide range of conditions, including rheumatic and neurological disorders [3].

Numerous therapies are aimed at improving UE functionality. In this context, whole‐body vibration (WBV) appears to represent a significant breakthrough in the field of rehabilitation [4]. WBV is a neuromuscular technique performed while patients stand on a vibrating platform. The mechanical vibration signals activate sensory receptors (muscle spindles), triggering reflex muscle activation like the tonic vibration reflex [5]. This therapy is typically administered in short periods, alternating with rest, often lasting less than 30 min. Effects of WBV depend on training posture, vibration frequency, and displacement amplitude, which vary depending on the devices and protocols used. Vibrations can be either nonstochastic (i.e., sinusoidal, nonrandom) or stochastic (i.e., nonsinusoidal, random), with the latter referred to as stochastic resonance therapy. While sinusoidal platforms can lead to the habituation of mechanoreceptor stimulation, stochastic platforms, which use three‐dimensional random movements, maintain continuous brain activation [6].

Although the use of WBV for muscle strengthening is well documented [7, 8], in recent years, WBV has gained attention in neurorehabilitation [9–12]. The mechanical stimulus produced by the vibrating platform is transmitted throughout the body, stimulating sensory receptors [13–15]. This somatosensory input appears to enhance brain plasticity by increasing corticospinal excitability [16] and activating the thalamus [17] and basal ganglia [6], as demonstrated in studies using functional magnetic resonance imaging, electroencephalography, heart rate variability, and electrocortical evoked potentials [16, 17]. These effects may also be attributed to the elevated concentrations of hormones and neurotransmitters such as growth hormone, testosterone, serotonin, and dopamine observed with WBV. Thus, while the exact mechanism of action remains unclear, it is thought to operate on multiple levels [18, 19].

Most research on neurological conditions has focused on the effects of WBV on postural control, balance, and gait. However, there is a notable gap in the literature regarding its potential benefits for UE functionality. Furthermore, the few studies that have been conducted—whether involving patients [20–23] or healthy participants [24–29]—predominantly use sinusoidal platforms and focus on muscle activation and strength rather than manual dexterity. These studies report mixed results, likely due to the diversity of protocols employed.

Additionally, research often applies WBV directly to the UE, as improvements have primarily been observed when the stimulus is applied close to the targeted area [25]. However, this approach frequently requires patients to adopt unergonomic and difficult‐to‐maintain positions [20, 21, 23, 27, 29]. Consequently, this preliminary pilot study is aimed at evaluating whether stochastic WBV can improve dexterity and manipulative capacity in healthy individuals using a conventional squat position over a short period (1 week).

If confirmed, the use of WBV could represent a significant advancement in hand functionality rehabilitation. Given that WBV is a therapy that can be performed at home in sessions typically lasting less than 10 min, it could be particularly beneficial for individuals with chronic neurological conditions who experience limited mobility or face barriers to accessing conventional therapy. It is essential to note that limited resources often restrict rehabilitation for neurological disorders to the subacute phase or periods of exacerbation. In this context, WBV could serve as a valuable alternative therapeutic option for such patients.

## 2. Methods

### 2.1. Design

This preliminary study is designed as a pilot study (Supporting Information (available here)).

### 2.2. Subjects

Thirty‐eight healthy adult (18–30 years old) volunteers were randomized using the Quick Calcs program (GraphPad Software Inc., La Jolla, California, United States) to the WBV group (*N* = 19; 6 men, 13 women) or the control group (*N* = 19; 6 men, 13 women).

They were recruited from Rey Juan Carlos University (Madrid, Spain).

To be included, the participants must give their informed consent. Each subject was instructed on the protocol, which was approved by the University’s Bioethics Committee (Code Number 040720255542025) according to the Helsinki declaration.

The exclusion criteria were any contraindication for WBV (pregnancy, recent fractures, malignancies, kidney stones, cardiac pacemaker, infectious disease, or recent thromboembolism). Also, subjects already treated with WBV were excluded.

### 2.3. Intervention

The subjects in the WBV group performed one series of five consecutive repetitions of 60‐s unsynchronized multidimensional WBV (Zeptoring, Scisen GmbH, Germany; mechanical vibration with frequency of 4 Hz and amplitude 3 mm) with a 1‐min pause between administrations, three times a week. The construction of this device is designed to perform a nonharmonious generation of oscillating movements in vertical and horizontal planes to prevent occurrences of resonance and habituation of receptors.

**Figure 1:**
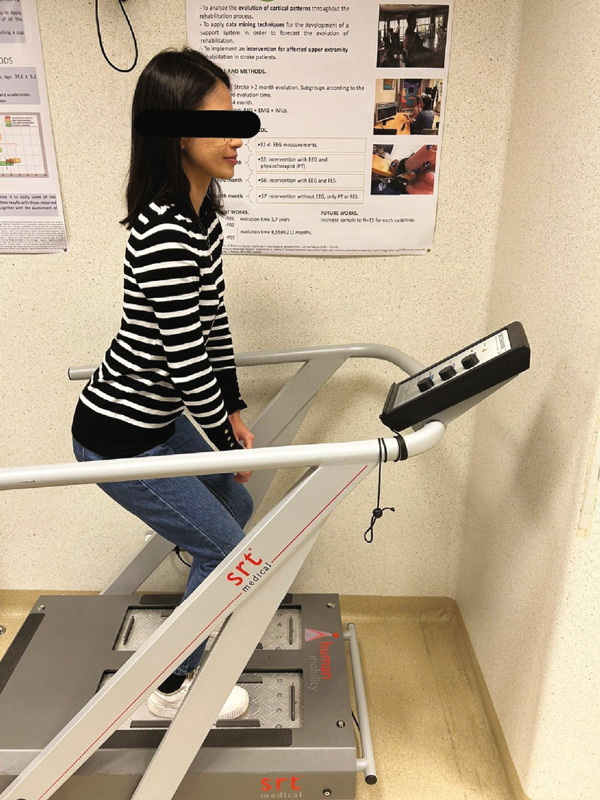
Stochastic multidimensional whole‐body vibrating platform.

During the intervention, subjects wore thin‐soled gymnastic‐type shoes, carrying out a squatting standing position with slight flexion at the hips, knees (40° flexion), and ankle joint on the vibrating platform (Figure 1).

The controls did not receive any training. Both groups were evaluated at baseline and 5 min after intervention, in the University’s Gait Analysis Laboratory.

Throughout the administration of all testing procedures, participants were continuously accompanied to prevent the occurrence of adverse effects. In the event that any such effects were observed, it was predetermined that the test would be immediately discontinued to ensure participant safety and to avoid compromising the integrity of the assessment.

### 2.4. Outcomes

Four outcome measures of the Purdue Pegboard test (PPT, Model 32020, Lafayette, United States): preferred hand (PH), nonpreferred hand (NPH), both hands (BH), and assembly (A), were performed.

The PH was determined by the hand freely chosen for writing (right hand for all cases).

The PPT was first developed by J. Tiffin in 1948 (Purdue University, Indiana, United States) to evaluate coordination and dexterity [30]. Since that time, this device has been used extensively in the selection of employees for manual jobs and in rehabilitation services to estimate hand function. PPT shows good test–retest reliability, with intraclass correlation coefficients of 0.90 [31].

**Figure 2:**
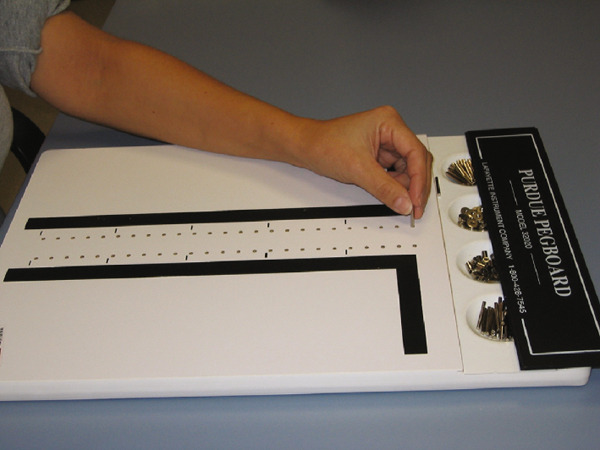
Purdue Pegboard test.

PPT measures two types of activities: gross movements of the hand, fingers, and arm and “fingertip” dexterity in an assembly task. It involves the sequential insertion of pegs and the assembly of pegs, collars, and washers (Figure 2). The number of pegs placed in the holes within 30 and 60 s was recorded for the first 3 inputs and assembly, respectively.

We considered one‐trial administration of PPT to be a sufficiently reliable assessment to use with healthy youngers [32].

A standardized form was developed to collect data on potential adverse events related to test administration, aiming to ensure procedural safety and to record any participant‐reported reactions or discomfort occurring during or following the assessment. All measurements were performed by the first author who was blind to group allocation.

### 2.5. Statistical Analysis

Quantitative variables were expressed as mean ± standard deviation (SD). The Kolmogorov–Smirnov test confirmed that the sample followed a normal distribution. Baseline characteristics of both groups were compared using Student’s *t*‐test. This test was also applied to identify significant differences in mean values before and after the intervention within the WBV and control groups. All analyses were conducted using SPSS Software, Version 26.0. Statistical significance was set at *p* < 0.05.

## 3. Results

Baseline characteristics of the study population are summarized in Table 1. The results of this study indicate that the majority of participants (68.4%) were women. There were no significant differences in the average age between the WBV and control groups (*p* = 0.62). The gender distribution was identical in both groups. At baseline, the WBV group showed similar performance to the control group in Purdue test measures.

**Table 1:** Baseline characteristics of the sample.

**Parameter**			**WBV vs. control**		
	**WBV**	**Control**	**MD**	**p**	**95% CI**
Age (years old)	21.05 (3.79)	20.47 (3.51)	0.57	0.621	From −1.25 to 1.32
PH	16.21 (1.81)	15.95 (2.04)	−0.263	0.677	From −1.53 to 1.01
NPH	14.47 (2.31)	14.74 (1.19)	0.263	0.663	From −0.95 to 1.48
BH	12.21 (1.71)	12.84 (1.61)	0.632	0.250	From −0.46 to 1.73
A	36.68 (6.25)	39.89 (3.84)	3.211	0.882	From −0.21 to 6.63

*Note:* Independent samples *t*‐test was used. *p* < 0.05 was considered significant. Data are expressed in mean (SD).

Abbreviations: A, assembly Purdue Pegboard test; BH, both hands Purdue Pegboard test; CI, confidence interval; MD, mean difference; NPH, nonpreferred hand Purdue Pegboard test; PH, preferred hand Purdue Pegboard test; WBV, whole‐body vibration.

A comparison of mean variable values between pre‐ and posttreatment for each group, conducted using the *t*‐test (Table 2), revealed significant differences where *p* values were considered significant at *p* < 0.05. In the WBV group, significant differences were observed for NPH, BH, and A scores posttreatment, while in the control group, a significant difference was found only for the A score.

**Table 2:** Within‐subjects’ analysis.

		**Mean (SD)**		**Baseline vs. immediately after intervention**		
		**Baseline**	**After**	**MD**	**p**	**95% CI**
PH	*WBV*	16.21 (1.81)	16.95 (1.68)	−0.737	0.176	From −1.84 to.36
	*Control*	15.95 (2.04)	17.11 (1.56)	−1.158	0.080	From −1.98 to −0.34
NPH	*WBV*	14.47 (2.31)	16.15 (1.57)	−1.684	**< 0.001** ^∗^	From −2.5 to −0.86
	*Control*	14.74 (1.19)	14.84 (1.46)	−0.105	0.716	From −0.70 to.49
BH	*WBV*	12.21 (1.71)	13.63 (1.64)	−1.421	**< 0.001** ^∗^	From −2.07 to −0.77
	*Control*	12.84 (1.61)	12.21 (0.97)	0.632	0.083	From −0.09 to 1.35
A	*WBV*	36.68 (6.25)	44.21 (5.34)	−7.526	**< 0.001** ^∗^	From −10.83 to −4.23
	*Control*	39.89 (3.84)	44.05 (4.5)	−4.158	**0.003** ^∗^	From −6.66 to −1.66

Abbreviations: A, assembly Purdue Pegboard test; BH, both hands Purdue Pegboard test; CI, confidence interval; MD, mean difference; NPH, nonpreferred hand Purdue Pegboard test; PH, preferred hand Purdue Pegboard test; WBV, whole‐body vibration.

^∗^Revealed by Student’s *t*‐test. *p* < 0.05 was considered significant.

The analysis of differences between the posttreatment means values of the measured variables in the two groups (Table 3) showed a significant advantage in favor of the WBV group for NPH and BH compared to the control group. Among all variables, the NPH showed the most substantial improvement, with an increase of approximately 12% (*p* < 0.02), while BH improved by 8% (*p* < 0.01).

**Table 3:** Group–time interaction analysis.

**Parameter**			**WBV vs. control**		
	**WBV**	**Control**	**MD**	**p**	**95% CI**
PH	16.95 (1.68)	17.11 (1.56)	0.158	0.766	From −0.91 to 1.23
NPH	16.15 (1.57)	14.84 (1.46)	−1.31	**0.011** ^∗^	From −2.32 to −0.32
BH	13.63 (1.64)	12.21 (0.97)	−1.42	**0.003** ^∗^	From −2.31 to −0.53
A	44.21 (5.34)	44.05 (4.50)	−0.15	0.922	From −3.41 to 3.10

*Note:* Data are expressed as mean (SD).

Abbreviations: A, assembly Purdue Pegboard test; BH, both hands Purdue Pegboard test; CI, confidence interval; MD, mean difference; NPH, nonpreferred hand Purdue Pegboard test; PH, preferred hand Purdue Pegboard test; WBV, whole‐body vibration.

^∗^ *p* of the Student *t*‐test. *p* < 0.05 was considered significant.

No adverse reactions were reported following the 5‐min mechanical vibration session, and no participants dropped out of the study.

## 4. Discussion

This study was conducted to evaluate the effect of WBV on manual skill performance. To the best of our knowledge, it is the first to analyze this effect using stochastic resonance WBV in a squatting position.

A few studies have analyzed the effects of WBV on UE function, but most of them focused on grip strength (using a dynamometer) or electromyographic activity [18, 23–29, 33]. Only the studies by Ahn et al. [20], Sade et al. [21], and Lee et al. [22] assessed hand motor dexterity. However, all of these studies used a sinusoidal vibrating platform.

Ahn et al. [20] used the Manual Function Test to assess motor function impairment in the affected UE of subacute stroke patients after 30 min of WBV (< 19 Hz) daily, 5 days a week, for 4 weeks, in addition to occupational therapy and conventional physical therapy. Each participant was seated in front of the vibrating platform in an unergonomic position (shoulders flexed at 90°, elbows slightly flexed, and trunk tilted forward to allow BH to rest on the platform). To minimize discomfort, participants were allowed to keep their palms slightly away from the vibrating platform. The authors observed improvements in the affected hand in both the WBV and control groups (up–down cycle), with greater improvements in the WBV group. Similar to our study, long‐term outcomes were not investigated. The device used was a sinusoidal model (Galileo 2000, Germany). Sade et al. [21] evaluated the effects of WBV on UE function in adult stroke patients using the Jebsen–Taylor Hand Function (JTHF) test after 3 weeks (2 sessions of 60 s, 5 times a week, at 35–40 Hz) of vibrotherapy using the Power Plate sinusoidal vibrating platform (Pro5, North America) plus conventional therapy. The patients’ affected limbs were positioned above the vibrating platform (elbow at 80° of flexion with the wrist in dorsiflexion). The authors found no significant difference in JTHFT scores between the WBV and control groups. Lee et al. [22] evaluated WBV training, alone or combined with task‐related training, for 4 weeks in poststroke patients. Their results showed a significantly greater increase in the Fugl‐Meyer scale of the affected hand with WBV training. There was also a significantly greater increase in the Wolf motor function test when task‐related training was added. The three previous trials included people with subacute and chronic conditions after stroke, so some recovery may have occurred spontaneously. Active movement of the upper limb enhances neuroplasticity and promotes motor recovery. However, as all patients also received conventional therapy, it is difficult to confirm that the observed improvements were solely due to WBV. In addition, the platforms used were sinusoidal and patients were required to place the affected arm directly on the vibrating platform.

Several studies have evaluated the position of the subject over a vibrating platform. Hussain et al. [23] observed the effects of WBV in weight‐bearing and nonweight‐bearing positions for the upper and lower extremities on hand grip strength in children with cerebral palsy. After 12 sessions, hand grip strength improved more in the weight‐bearing position for both the upper and lower extremities simultaneously. Santos et al. [25] applied WBV (with a sinusoidal platform, at 45 Hz) to 19 healthy women in a modified push‐up and half‐squat position. They studied hand grip strength and concluded that the distance of the stimulus and the position on the vibrating platform influence the maximum muscular strength due to neuromuscular modifications of the hands in healthy women. In the half‐squat position, the vibratory stimulus reaching the upper limbs is insufficient to promote a more efficient contraction and consequent increase in strength. Muscles that are not directly exposed to mechanical vibration do not show the same increase in performance as the vibrated muscle. Other studies in healthy volunteers show similar results [27]. In contrast, Morel et al. [28, 29] found no significant main effect of hand grip strength measurement between push‐up and squat conditions in healthy soldiers.

Our results show that brief WBV treatment leads to improvements in manual dexterity in healthy subjects using a stochastic‐random platform with participants seated in a comfortable position (squat). We observed a significant difference in pretreatment NPH, BH, and A scores in the WBV group but not in the dominant hand. Cochrane and Hawke [34] also found no improvement in the dominant hand performance of climbers. One possible explanation is that the dominant hand may have less room for improvement because its routine use already requires maximum muscular effort.

In physics, stochastic resonance is a nonlinear cooperative effect in which a weak periodic mechanical stimulus induces large‐scale fluctuations, resulting in the amplification of the weak component [6]. This phenomenon has been observed in living systems, where weak signals can be amplified by adding an optimal level of external random signals to individual sensory neurons. Stochastic resonance allows significant improvements in the detection of weak periodic signals [35]. To the best of our knowledge, this is the first study to investigate the effect of 1 week of low‐frequency (4 Hz) stochastic resonance on manual dexterity in healthy individuals in a squatting position.

Unsynchronized multidimensional devices have been used in Parkinson’s disease [36] and multiple sclerosis [37] with improvement in clinical symptoms such as bradykinesia, imbalance, and gait disturbance, which may mean that WBV has stimulatory effects on the human central nervous system (CNS), potentially explaining why stochastic WBV is effective in complex neurological conditions [6]. However, the mechanism of action of WBV remains unknown.

Improvements have been speculated to be associated with increased neural excitation, possibly achieved through modulation at both the spinal and supraspinal levels of the CNS. Spinal modulation refers to involuntary reflex muscle activation, whereas the supraspinal level involves brain structures responsible for voluntary movement control [16]. At the supraspinal level, sensory input is integrated in both subcortical and cortical areas of the CNS. These responses tend to last longer than reflex activity and involve planned, situationally adapted, and much more specific muscle responses. It has been shown that WBV during squat exercise leads to a significant increase in motor evoked potentials compared to no WBV, suggesting that mechanical vibration increases motor cortex excitability and corticospinal facilitation, along with intracortical modulation involving increased intracortical inhibition and reduced intracortical facilitation. It is possible that cortical and subcortical facilitation accompanied by reduced spinal excitability may occur after an acute bout of WBV, indicating improved control of voluntary movement [13, 14, 16, 38].

However, the benefits observed with WBV in CNS disorders suggest that other mechanisms are also at play. Sant et al. [17] demonstrated that brief, low‐frequency spinal mechanoreceptor activation inhibited the firing rate of ventral tegmental area GABA neurons while increasing the firing rate of central tegmental dopamine neurons. Other studies have reported increases in cerebral dopamine, norepinephrine, serotonin, and cholinergic activity. Meanwhile, Kaut et al. [6] described an increase in movement associated with greater caudate nucleus activity after WBV. This may be explained by the fact that the basal ganglia are crucial for the execution of voluntary movements and modulate the activity of motor regions in the cortex. In particular, the caudate nucleus increases the speed and precision of movements directed at specific objects [39].

### 4.1. Study Limitations

There are several limitations to this study. First, it is a preliminary pilot study with a small sample size and limited statistical power. Therefore, the reported findings should be considered preliminary and require further evaluation of larger samples. Second, we did not correlate the functional assessment of manual dexterity with potential cerebral activation. Third, although there is evidence that the acute residual effects of WBV last only a few minutes after mechanical vibration training [28], we did not investigate this aspect.

## 5. Conclusions

A key strength of this study is the use of a stochastic resonance WBV platform in a squat position, which differs from the nonstochastic platforms commonly used in previous research. Furthermore, this is one of the first investigations to assess manual dexterity—beyond grip strength or muscle activity—under these specific mechanical vibration conditions. It is important to highlight that manipulative dexterity is essential for the performance of activities of daily living. Therefore, the results obtained support the therapeutic potential of this type of intervention. In this regard, stochastic resonance vibration platforms could represent an effective treatment option, as they provide considerable functional benefits within a relatively short period of time. In conclusion, this study suggests that stochastic resonance WBV in a squatting position may improve manual dexterity in a short period of time. These findings support its potential as a complementary therapeutic strategy, particularly in contexts with limited resources or high demand. Further research is needed to confirm its applicability in clinical populations.

## 6. Implications for Rehabilitation

- A brief WBV (5 min per day, three times) intervention is enough for enhancing manual ability performance.
- WBV therapeutic could be a beneficial addition in diseases with hand ability impairment.
- Fatigue or lack of interest can reduce the feasibility of extended rehabilitation programs. WBV therapeutic could be an alternative, especially in people with a poor functional capacity.
- It can easily be made accessible to large populations due to the minimal training required to administer.

## Disclosure

All authors read and approved the final version of the manuscript.

## Conflicts of Interest

The authors declare no conflicts of interest.

## Author Contributions

I.M.A.D. and R.M.M.P. contributed in investigation. I.M.A.D. and F.M.R. designed the experimental protocol. R.M.M.P. and Á.A.R. recruited the patients. Á.A.R. performed the assessments. M.G.A. analyzed the data. I.M.A.D., R.M.M.P., Á.A.R., M.G.A., and F.M.R. wrote the manuscript.

## Funding

No funding was received for this manuscript.

## Supporting information


**Supporting Information** Additional supporting information can be found online in the Supporting Information section. CONSORT Harms 2022 integrated into CONSORT 2010 items checklist of information to include when reporting a randomized trial.

## Data Availability

The data that support the findings of this study are available from the corresponding author upon reasonable request.
